# Serum Metabolomics Reveals Distinct Profiles during Ischemia and Reperfusion in a Porcine Model of Myocardial Ischemia–Reperfusion

**DOI:** 10.3390/ijms23126711

**Published:** 2022-06-16

**Authors:** Eric Goetzman, Zhenwei Gong, Dhivyaa Rajasundaram, Ishan Muzumdar, Traci Goodchild, David Lefer, Radhika Muzumdar

**Affiliations:** 1 Children’s Hospital of Pittsburgh of UPMC, Pittsburgh, PA 15260, USA; eric.goetzman@chp.edu (E.G.); zhenwei.gong@chp.edu (Z.G.); dhivyaa.rajasundaram@chp.edu (D.R.); 2 Department of Pediatrics, University of Pittsburgh, Pittsburgh, PA 15260, USA; 3 School of Undergraduate Study, Penn State University, State College, PA 16802, USA; imuzumdar@gmail.com; 4 Cardiovascular Center of Excellence, Louisiana State University Health Sciences Center, New Orleans, LA 70112, USA; tgoodc@lsuhsc.edu (T.G.); dlefe1@lsuhsc.edu (D.L.)

**Keywords:** myocardial ischemia–reperfusion, metabolomics, mitochondrial dysfunction, serum biomarkers

## Abstract

Acute myocardial infarction (MI) is one of the leading causes of death worldwide. Early identification of ischemia and establishing reperfusion remain cornerstones in the treatment of MI, as mortality and morbidity can be significantly reduced by establishing reperfusion to the affected areas. The aim of the current study was to investigate the metabolomic changes in the serum in a swine model of MI induced by ischemia and reperfusion (I/R) injury, and to identify circulating metabolomic biomarkers for myocardial injury at different phases. Female Yucatan minipigs were subjected to 60 min of ischemia followed by reperfusion, and serum samples were collected at baseline, 60 min of ischemia, 4 h of reperfusion, and 24 h of reperfusion. Circulating metabolites were analyzed using an untargeted metabolomic approach. A bioinformatic approach revealed that serum metabolites show distinct profiles during ischemia and during early and late reperfusion. Some notable changes during ischemia include accumulation of metabolites that indicate impaired mitochondrial function and N-terminally modified amino acids. Changes in branched-chain amino-acid metabolites were noted during early reperfusion, while bile acid pathway derivatives and intermediates predominated in the late reperfusion phases. This indicates a potential for such an approach toward identification of the distinct phases of ischemia and reperfusion in clinical situations.

## 1. Introduction

Coronary artery disease is a major cause of morbidity and mortality in the world. In the US, heart disease remains the number one cause of death across all ages and accounts for >25% of the deaths in people 65 years and older. (https://www.cdc.gov/nchs/data/nvsr/nvsr70/nvsr70-09-508.pdf) (accessed on 1 March 2022). According to the American Heart Association (AHA), it is estimated that an American will have a heart attack approximately every 40 s [1]. In an analysis of 23 published studies in which 14,211 patients were followed prospectively following a myocardial infarction (MI), it was reported that, on average, 23% of patients died before reaching the hospital following the first MI, and another 13% died during hospital admission [2]. Causes of death in these patients include ventricular arrythmias, stroke, cardiac rupture, and acute cardiac failure. Those who survive an MI typically exhibit subsequent myocardial remodeling through excessive myofibroblast proliferation, extracellular matrix deposition, and cardiac hypertrophy, causing significant morbidity and mortality [3]. Post-myocardial infarction heart failure is common and is strongly associated with mortality in patients. In the elderly patients who underwent percutaneous coronary intervention for ST-elevation myocardial infarction (STEMI), the 8 year mortality of patients was as high as 49% [4].

The goal in the treatment of myocardial infarction is to preserve the myocardium. Current treatment paradigms involve restoring blood flow (reperfusion) as quickly as possible to limit permanent ischemic damage to the cardiac tissue. Time is a critical factor in the prognosis, and “time to table” is an important concept in management of acute coronary vascular events. An earlier diagnosis and prompt restoration of circulation are crucial for a better prognosis. While effective in this regard, reperfusion itself can lead to significant injury of myocardium. Hence, there exists a continued need to identify early markers of coronary events and effective treatment options.

Deprivation of supply of oxygen and nutrition results in biochemical and metabolic changes in the myocardium. In a well-perfused heart, almost all (>95%) ATP formation is derived from oxidative phosphorylation in the mitochondria. Approximately 60–90% of the acetyl CoA that enters the TCA cycle is derived from fatty-acid oxidation (FAO), while 10–40% is from oxidation of pyruvate derived either from glycolysis or from oxidation of lactate. In the ischemic myocardium, there is an increase in anaerobic glycolysis and, therefore, lactate production, in an attempt to meet the energy needs for contraction [5]. Glycolytic ATP is critical in the post-ischemic/reperfused (IR) myocardium for optimal diastolic relaxation [5]. There are inherent advantages in carbohydrate utilization under these conditions as glycolysis is quicker and uses less oxygen than FAO. During reperfusion, there is increased free fatty acid (FFA) supply to the heart and a reliance on FAO, which exacerbates myocardial injury through generation of free radicals and increased oxidative stress [6]. FFAs increase calcium, and the effort at Ca cycling to maintain homeostasis increases the rate of ATP hydrolysis, leading to “wastage” of ATP [5]. These are some of the phase-specific changes that occur in the ischemic and reperfused myocardium. Although these changes are intended to maintain cardiac function and meet the myocardium’s metabolic needs, they could have adverse consequences.

Many metabolic approaches have been tried in the management of myocardial ischemia with variable success. Increasing glucose metabolism through infusion of insulin, pyruvate infusion, use of glucagon-like peptide-1 (GLP-1)-based therapies, and AMP-activated protein kinase (AMPK) activation using metformin and cardiac-specific gain of function of glucose transporter GLUT 1 have all been shown to decrease infarct size in IR models through stimulation of myocardial glucose metabolism [7,8] and suppression of FFA release from adipocytes [5,9,10]. In the glucose–insulin–potassium (GIK) clinical trials, even though there were no changes in 30-day mortality or progression to MI, there was a decrease in infarct size and in-house mortality, as well as an improvement in composite outcome following cardiac arrest [11]. Infusion of insulin improved LV ejection fraction in patients with LV dysfunction following ischemic heart disease [12]. Dichloroacetate (DCA), a compound that increases glucose oxidation by stimulating pyruvate dehydrogenase (PDH) through inhibition of pyruvate dehydrogenase kinase (PDK) activity, has been shown to decrease infarct size and improve cardiac efficiency in IR, potentially by switching the heart toward the more efficient fuel, i.e., glucose [13]. Similarly, approaches to decrease FAO with either enzyme inhibitors or genetic approaches have been shown to decrease infarct size [14]. Partial inhibitors of myocardial FAO have been shown to be effective in animal models of IR, as well as in patients with chronic stable angina [15], through increased pyruvate oxidation and decreased lactate accumulation. In addition, the contractile performance of the heart is better with glucose oxidation compared to fatty acids at a given myocardial oxygen consumption. Increased plasma FFA concentration has been associated with a decrease in cardiac mechanical efficiency in humans [16], pigs [17], and dogs [18]. The FAO inhibitor ranolazine has been shown to decrease myocardial oxygen consumption and improve mechanical efficiency of the LV in dogs [18]. These studies highlight a potential benefit in decreasing FAO in conditions of IR to improve cardiac survival and function post MI/R. While it is clear that therapeutic options to switch substrate metabolism could improve cardiac survival and function, many pharmacological agents targeting these mechanisms, such as dichloroacetate or etomoxir, albeit very effective in animal models, have not translated to the clinic due to side-effects [19,20,21]. In addition to metabolic changes, both ischemia and reperfusion injury are associated with ROS-related damage to the mitochondrial respiratory chain machinery and other metabolic components. This leads to further ROS production and bioenergetic decline, which ultimately leads to cell death [22,23]. While newer options are being evaluated for their ability to offer cardioprotection, early diagnosis remains critical to initiate available modalities to improve the coronary circulation, minimize the damage, and prevent long-term morbidity.

Could we leverage the changes in metabolites toward timely diagnosis? While some metabolite changes are the result of fuel shortage and metabolic adaptations, others reflect processes including cell damage, inflammation, and other compensatory responses. Using a metabolomics-based approach to study coordinated changes in metabolic pathways during the various phases of MI/R such as ischemia, early or late reperfusion may identify early biomarkers unique to the specific phases. Indeed, many animal studies have focused on the specific changes in the myocardium in these various phases. While those studies are extremely informative, the tissues are not available during routine clinical care of patients. Serum metabolomics offers the ability to map all the changes in the network in circulation and could provide important clinical, translatable insights into potential key changes that can serve as true biomarkers. Therefore, we performed untargeted metabolomics in the serum at baseline, at end of ischemia, and at early and late reperfusion in a porcine model of myocardial ischemia–reperfusion.

## 2. Methods

### 2.1. Swine MI/R Model

MI/R injury on mini swine was performed using the Consortium for Preclinical Assessment of Cardioprotective Therapies (CAESAR) model as described previously [24,25,26]. Myocardial ischemia was induced by angioplasty balloon occlusion of the proximal LAD coronary artery for 60 min, followed by balloon deflation and confirmed reperfusion via coronary angiography that demonstrated complete LAD patency. Serum samples were collected at baseline, after 60 min of ischemia, and at 4 and 24 h after reperfusion, before immediately storing them at −80 °C until further analysis. All procedures were approved by the Institutional Animal Care and Use Committee of Louisiana State University Health Science Center.

### 2.2. Metabolomic Profiling

Pig serum samples were snap-frozen in liquid nitrogen and maintained at −80 °C until analysis by Metabolon, Inc. (Morrisville, NC, USA) as described [27]. To prevent accidental sample misidentification, the Metabolon Laboratory Information Management System (LIMS) was utilized. Each sample received a unique identifier through which it was tracked throughout sample handling and mass spectrometry analysis. Samples were prepped for analysis using an automated system (MicroLab STAR system, Hamilton, Reno, NV, USA). First, recovery standards were added, and then the serum samples were deproteinized by methanol precipitation. The extract was divided into five portions: two for analysis by reverse-phase ultra-performance liquid (UPLC)–MS/MS with positive ion mode electrospray ionization (ESI), one for analysis by reverse-phase UPLC–MS/MS with negative ion mode ESI, one for analysis by hydrophilic interaction (HILIC) UPLC–MS/MS with negative ion mode ESI, and one stored as backup material. The purpose of the four analytical runs under different conditions was to ensure unbiased, maximal coverage across different classes of metabolites.

Metabolon utilizes several layers of quality control as described previously [27]. In addition to the recovery standards added to each sample during processing/extraction, several quality control samples were run concomitantly with the experimental samples: (1) small volumes of the experimental samples were pooled and used as a technical replicate throughout the analysis, (2) extracted water samples served as process blanks, and (3) a cocktail of standards, chosen because they do not interfere with the measurement of desired metabolites, were added to each sample. These standards allowed Metabolon to monitor performance of the mass spectrometry instruments via calculation of the instrument variability parameter. For the present study, the instrument variability was 7%, which falls into the acceptable range dictated by the Metabolon quality control rubric.

All data for this study were acquired using a Waters ACQUITY UPLC and a Thermo Scientific Q-Exactive high resolution/accurate mass spectrometer. This instrument is equipped with a heated electrospray ionization source and an Orbitrap mass analyzer operated at 35,000 mass resolution. As mentioned above, each serum sample extract was divided into four aliquots to achieve maximal coverage of metabolite classes with differing properties. One aliquot was chromatographically separated on a C18 column (Waters UPLC BEH C18, 2.1 × 100 mm, 1.7 µm) with an elution gradient optimized for hydrophilic compounds. A second aliquot was also chromatographed on a C18 column but with elution conditions favoring hydrophobic compounds. The third aliquot was analyzed using basic negative ion optimized conditions, and the fourth aliquot was analyzed via negative ionization following elution from a HILIC column (Waters UPLC BEH Amide, 2.1 × 150 mm, 1.7 µm). The analysis alternated between MS and data-dependent MSn scans using dynamic exclusion, covering 70–1000 *m*/*z*. The resulting spectra were extracted, peak-identified, and QC-processed using Metabolon’s proprietary software (Metabolon Inc, Morrisville, NC, USA). The data preprocessing pipeline, designed to ensure high-quality datasets for subsequent statistical analysis, was previously described [27]. Briefly, metabolites in the pig serum samples were identified by matching to Metabolon’s 5200+ chemical library containing purified standards or recurrent unknown entities. This library contains retention time/index (RI), mass-to-charge ratio (*m*/*z*), and chromatographic data (including MS/MS spectral data) for each metabolite. Metabolite identifications were made by matching the retention index, mass +/− 10 ppm, and MS/MS forward and reverse scores between the experimental data and library standards. Proprietary software was used to confirm the consistency of peak identification. In the present study, a total of 736 metabolites were identified across the set of pig serum samples. Following metabolite identification, each peak was quantified by calculating the area under the curve.

### 2.3. Workflow and Analyses of Metabolome Data

The workflow and analyses of metabolome data are summarized in Supplementary Figure S1. In brief, after imputation of missing values and removing metabolite rows that contained all zero values, downstream analyses were carried out as detailed below.

**sPLS-DA:** The relationships of the metabolome profiles between the different ischemia–reperfusion groups/models were assessed using both principal component analysis (PCA) and sparse partial least squares discriminant analysis (sPLS-DA). The analysis was conducted using mixOmics R package [28]. In order to identify the metabolic signatures that distinguish the phenotypes, sPLS-DA was used, and the model performance was assessed using leave-one-out cross-validation. Different visualizations such as the sample plots with 95% confidence ellipses were used to observe the strong stratification of the samples, and the contribution of the metabolites to the different components that discriminate the phenotype was assessed using variable importance plots and clustered image maps.

**Co-expression analyses:** Co-expression networks were constructed for the porcine models of myocardial ischemia–reperfusion using the Bioconductor WGCNA package [29] with the following parameters: networkType  =  ‘signed’, softPower  =  9 or 10, minModuleSize  =  30, and deep Split  =  2. Briefly, signed networks were constructed, and, for each metabolite in the matrix, a pairwise Pearson correlation coefficient was calculated, followed by computing the adjacency matrix which was then transformed into a network of topological overlap. The topological overlap matrix was hierarchically clustered to identify highly co-expressed metabolites and grouped into modules by the dynamic tree cut algorithm. The modules were summarized by a module eigengene (ME) and were assigned colors as indicated in the horizontal bar of the dendrogram, and the module stability was tested using the module preservation function in order to provide rigorous quantitative statistics of module preservation. After co-expressed metabolite module identification, we linked the modular metabolite expression with the phenotypes that helped us discover module signatures functioning under a specific condition of the ischemia–reperfusion. Functional annotation of the network modules was performed using the enrichment analysis feature from MetaboAnalyst 5.0 [30] with *Homo sapiens* (SMPDB) as the pathway library of choice.

**Statistical analysis:** The in vivo studies were performed with seven animals. All values shown are presented as the means ± SEM. An independent two-tailed *t*-test was used when comparing two groups between baseline and ischemia phase only. One-way ANOVA was used when more than three conditions were involved. For each statistically significant F-value observed for the main effect or interaction, a two-tailed post hoc test (Tukey’s) was applied to determine individual differences between means. The difference was considered to be statistically significant when *p* ≤ 0.05.

## 3. Results and Discussion

### 3.1. The Stages of IR Injury Show Distinct Serum Metabolite Profiles

**Figure 1:**
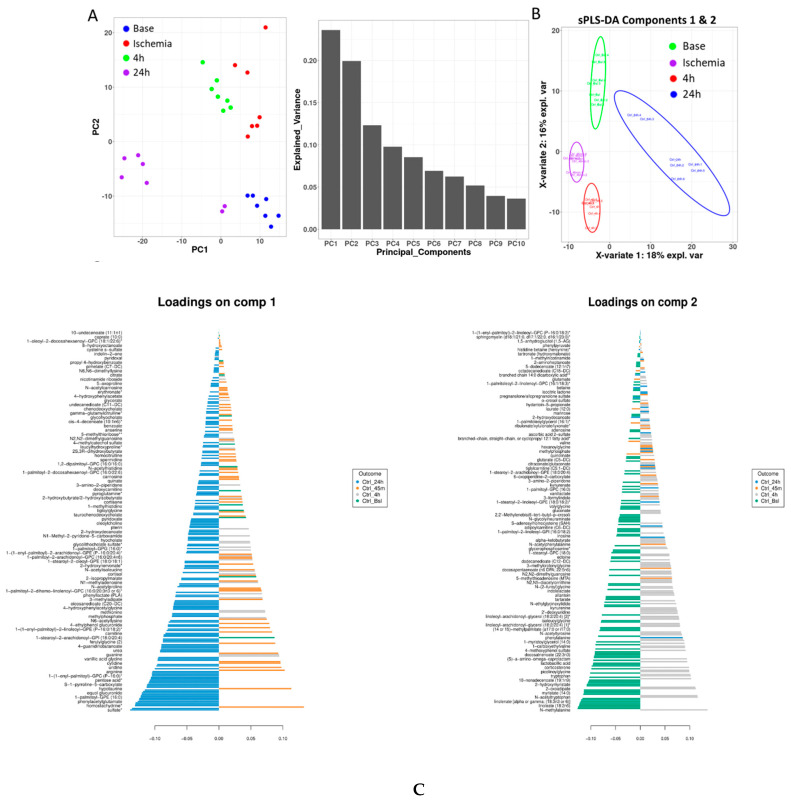
**PCA and sPLSDA:** The sample plots of the implemented statistical models using (**A**) PCA and (**B**) sPLS-DA significantly distinguish the four phases. Principal components 1 and 2 depict 18% and 16% of the variation, respectively. The sample plot depicts the first two sPLS-DA components with 95% confidence ellipse plots. (**C**) The variable importance in projection (VIP) plots represent the contribution of metabolites that significantly separate/discriminate the different phases on the first two components. The length of the bar represents the importance of each metabolite, and color coding depicts the phase in which it was most abundant with the importance ranked from bottom to top. Variables with the same distance from 0 with similar positions are positively correlated, and the ones in the opposite direction are negatively correlated.

Principal component analysis (PCA) was performed to illustrate the metabolite alterations as an effect of time (phase) in pigs using serum samples. The unbiased PCA analysis exhibited a high degree of individual variation and showed the samples profiled at baseline, during ischemia, and at two recovery points as unique and distinct clusters, confirming that the sample size was sufficient to conduct further downstream analysis. To identify the time-phase-associated metabolites, we performed a supervised sparse partial least squares discriminant analysis (sPLSDA), which also confirmed the distinct clustering of the samples, warranting a noticeable metabolic difference that can be attributed to the discriminated effect of time. The score plot of the sample shows that the PC1 and PC2 values explained 19% and 16% of the data variation, respectively, highlighting the metabolic alterations among the base, ischemia, 4 h and 24 h samples (Figure 1). We employed complementary measures such as repeated cross-fold validation to avoid overfitting the data, and the results are consistent with a string characteristic metabolite signal that distinguishes the different phases. Variable importance in projection (VIP) scores were calculated to identify the most discriminating metabolites between the time (phase)-separated samples.

**Figure 2:**
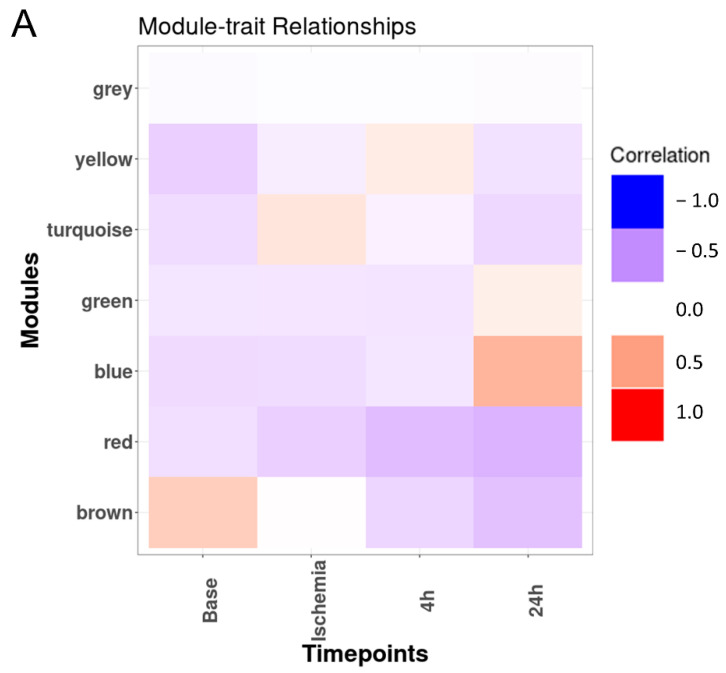

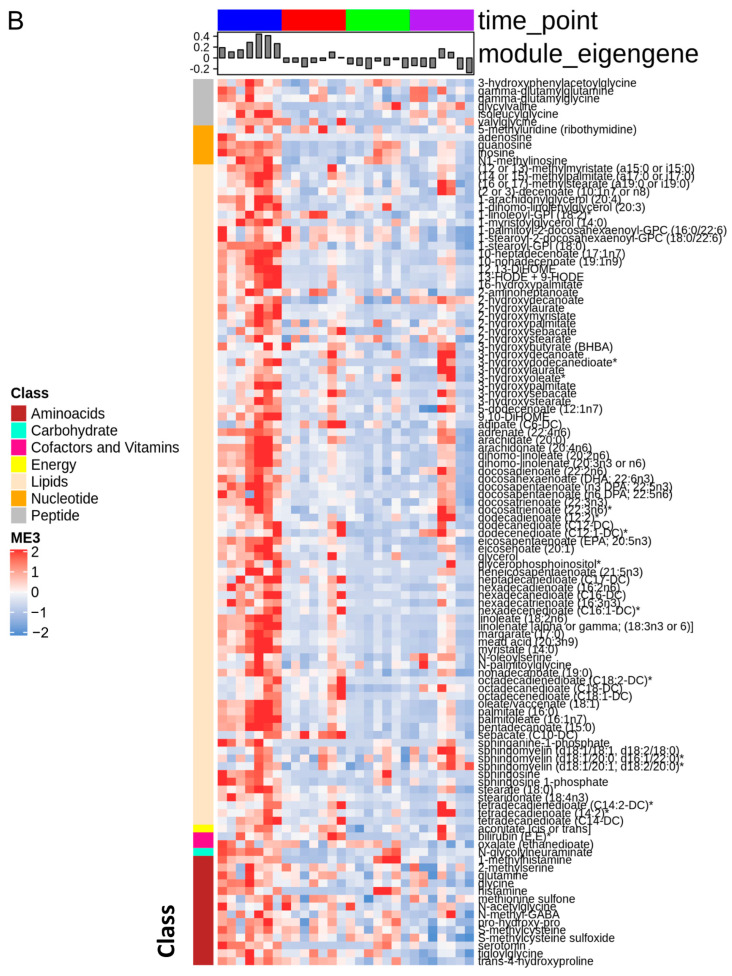

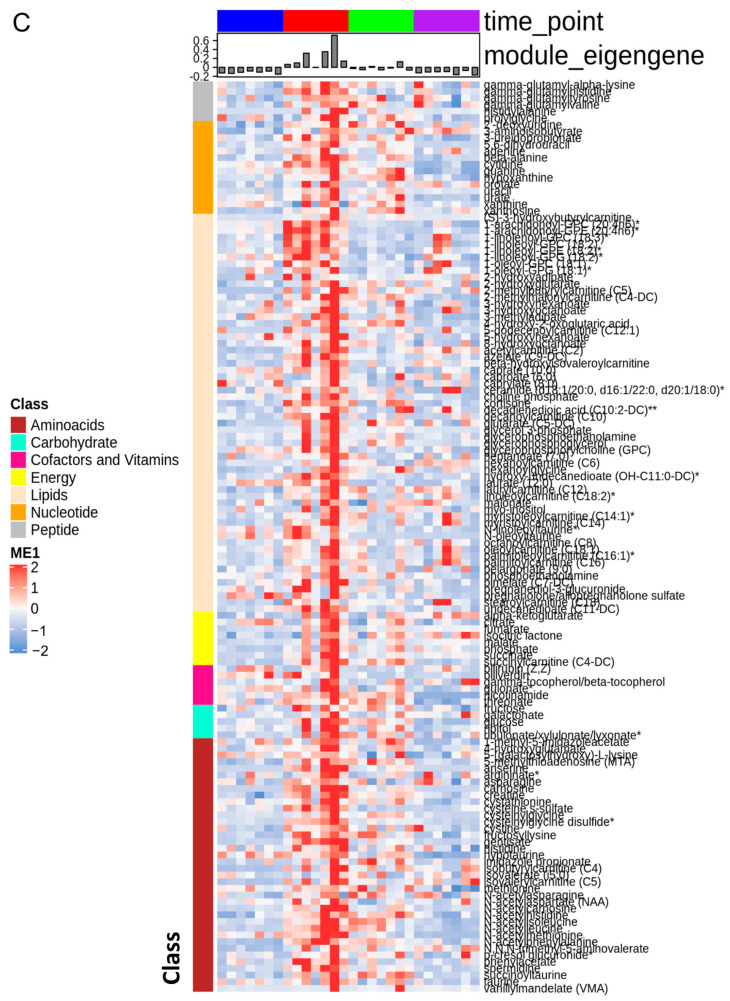

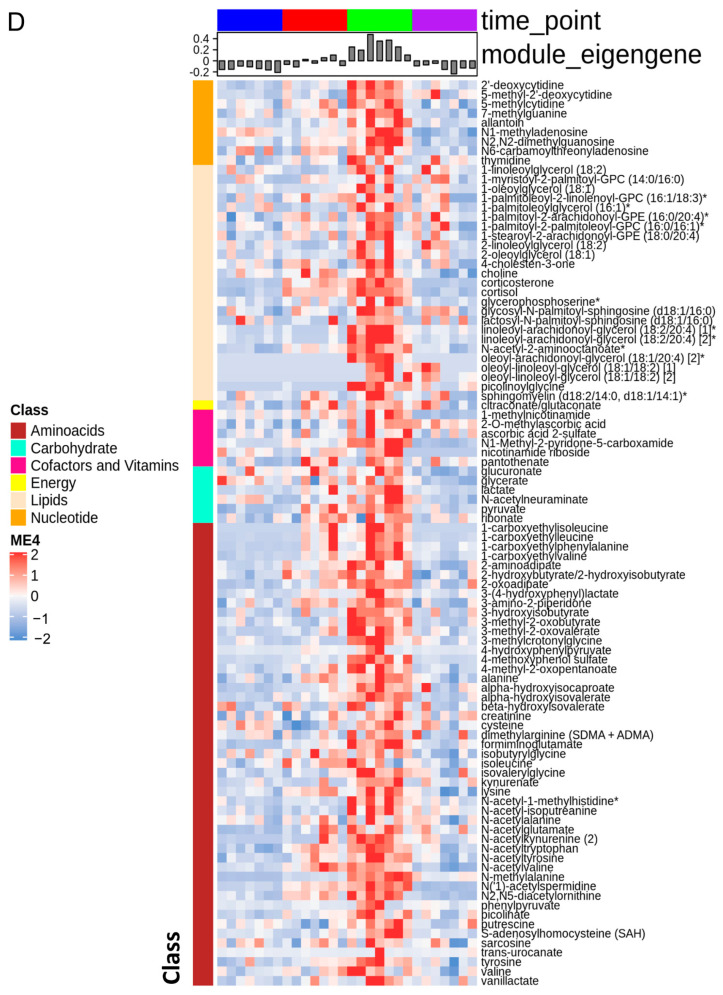

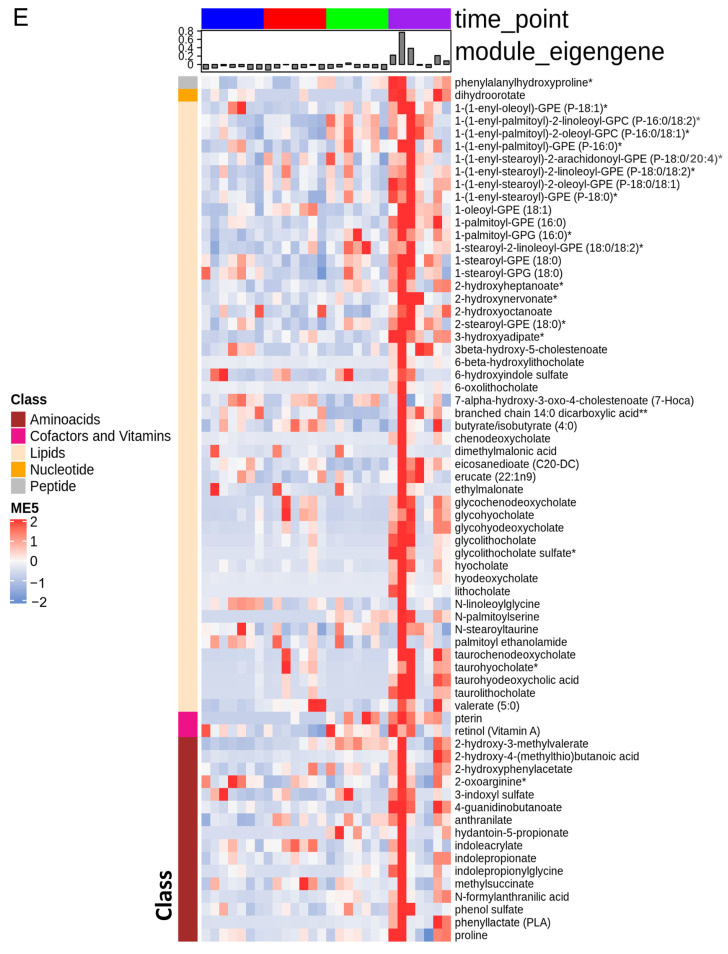
**Co-expression heatmaps:** (**A**) Heatmap of module–trait relationship depicting the correlation between module eigen metabolites and timepoints. The module eigen metabolite is defined as the first principal component of a given module and considered a representative of the metabolite profile in a module. The grid is color-coded by correlation according to the color bar of the correlation: red for positive and blue for negative correlation. (**B**–**E**) Heatmaps of the co-expressed metabolites in the ME3, ME1, ME4, and ME5 modules depicting the baseline, ischemia, 4 h reperfusion, and 24 h reperfusion phases, respectively, in the serum samples. Eigengene metabolite abundance at different phases is represented as the column annotation, while the row annotation corresponds to the different classes of metabolites. The metabolite values are centered and scaled, and low abundance is indicated by blue, while high abundance is indicated by red. * Indicates a compound that has not been confirmed based on a standard during mass spectrometry, but there is high confidence in its identity. ** Indicates a compound for which a standard is not available, but Metabolon is reasonably confident in its identity or the information provided.

Consequently, we used the WGCNA package to construct co-expression networks using the metabolite profiles of the four groups of samples. We selected the power value, which is one of the critical parameters, such that the independence and average connectivity was higher. A total of seven modules containing all metabolites were identified, and the metabolites of each module are demonstrated as a clustering dendrogram that depicts the topological overlap, together with module assigned colors and numbers (Figure 2A). The clinical traits of our samples included samples profiled at baseline, during ischemia, and at two recovery points (4 h and 24 h), and we calculated the correlation coefficients between the modules and the traits. The results elucidated that the brown, turquoise, yellow, and blue modules displayed highest correlation with baseline, ischemia, 4 h reperfusion, and 24 h reperfusion traits (Figure 2A). Figure 2B–E demonstrate the heatmaps of the co-expressed metabolites in the ME3, ME1, ME4, and ME5 modules depicting the base, ischemia, and 4 h and 24 h serum samples, respectively. In these heatmaps, the rows correspond to the different metabolites, while the column annotation indicates eigengene metabolite abundance at different phases. The metabolite values are centered and scaled, and low abundance is indicated by blue, while high abundance is indicated by red.

### 3.2. Ischemia Induces Changes in Metabolites That Indicate Impaired Mitochondrial Function

In order to confirm the biological themes of the metabolites associated with the trait-related modules and find the underlying functional enrichment, we performed KEGG enrichment analysis using Metaboanalyst 5.0.

**Figure 3:**
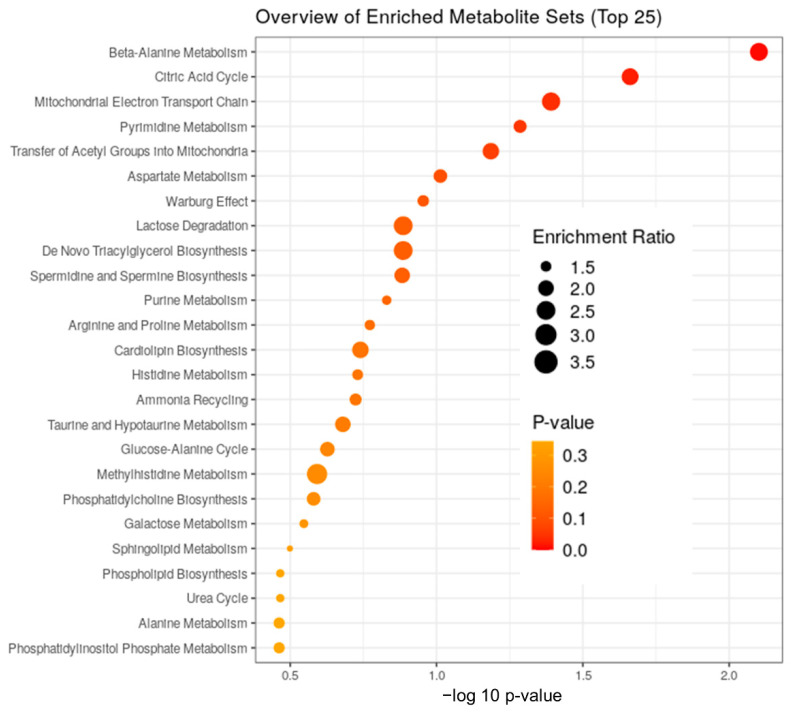
**Functional analysis plots:** Functional analysis for metabolites from the specific modules was performed to look for enrichment of sets of functionally related metabolites using the Small Molecule Pathway Database (SM bvcmouPDB) annotation system from MetaboAnalyst. Enrichment ratio is computed by Hits/Expected, where Hits = observed hits, and Expected = expected hits.

**Figure 4:**
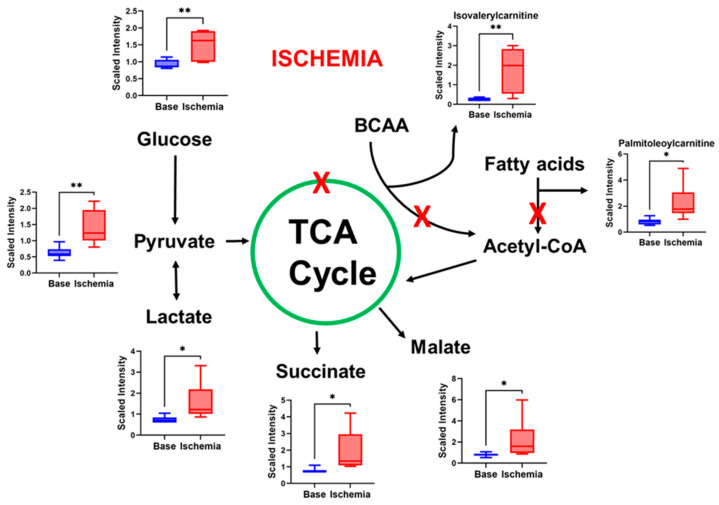
**Ischemia induces changes in metabolites that indicate impaired mitochondrial function.** TCA cycle intermediates, acyl carnitines, and BCAA acyl accumulate in the serum during ischemia. Shown are representative metabolites from the acylcarnitine and BCAA pathways (see also Supplementary Table S1). A two-tailed *t*-test was used to determine significance. *N* = 7; * *p* < 0.05 and ** *p* < 0.01.

A total of 138 metabolites were significantly changed during ischemia compared to all other phases. Their enrichment ratios, analyzed using the SMPDB pathway (Figure 3) and heatmap analysis (Figure 2B), show that some of the major changes noted during ischemia included TCA cycle intermediates, acyl carnitines, polyamine pathway, branched-chain amino-acid metabolites, and modified amino acids (Figure 4). In addition, there was a significant decrease in polyunsaturated fatty acids.

Blood glucose levels increased during the early phase of acute MI. This could be a consequence of stress; however, it could also serve as a major energy source for the heart. During myocardial ischemia, an increase in the rate of glycolysis and a switch to lactate production have been described. Indeed, our data showed increased levels of pyruvate and lactate in the serum during ischemia and early reperfusion phases. Citrate levels were lower (data not shown), and this could increase glycolysis because citrate is a negative regulator of glycolysis by inhibiting phosphofructokinase [31].

TCA cycle intermediates including isocitric lactone, alpha-ketoglutarate, succinate, fumarate, and malate were elevated during ischemia, while citrate, the entry point of the TCA cycle, was lower. This suggests mitochondrial stunning and impaired functioning of TCA cycle during ischemia. In addition to the TCA cycle, mitochondria are the site of FAO and branched-chain amino-acid (BCAA) oxidation (valine, leucine, and isoleucine). We observed an increase in BCAA metabolites and acyl carnitines, a well-known indicator of impaired FAO. In fact, TCA cycle intermediates and acyl carnitines accounted for ~23% of the changes in metabolites during ischemia. BCAAs promote unfavorable substrate switches especially in the ischemic or reperfused myocardium; they suppress cardiac glucose oxidation via inhibiting PDH activity and increase FAO through peroxisome proliferator-activated receptor (PPAR) alpha [32]. While specific impairment of BCAA oxidation could be a feature of the specific phases of the insult as noted in our study, the severity and/or persistence of this abnormality can lead to long term morbidity. Indeed, Uddin et al. showed that BCAA metabolites are higher in the hearts of subjects with dilated cardiomyopathy [33]. Furthermore, stimulation of BCAA oxidation improves the percentage ejection fraction (% EF) without altering cardiac hypertrophy. To what extent the source of serum BCAA metabolites in our model was the cardiac muscle versus other tissue needs to be clarified in future studies.

### 3.3. FFAs Related to Energy Metabolism Are Reduced by Ischemia

Ischemia–reperfusion affected lipid species differently across different lipid classes. The primary fatty-acid species in the diet, which are, thus, stored in adipose tissues, are palmitic (C_16_), palmitoleic (C_16:1_), stearic (C_18_), and oleic (C_18:1_) acids. These four fatty-acid species constitute the majority of fatty acids circulating in the body for the purpose of mitochondrial energy generation. Serum levels of all four fatty-acid species declined during the ischemic period and remained suppressed until 24 h post reperfusion Table 1)). At the same time, the corresponding acylcarnitine species, which are exported from mitochondria whenever β-oxidation flux is impaired, were increased during ischemia (Figure 4).

Specifically, changes in free monocarboxylic fatty acids and dicarboxylic acids were noted in the ischemia phase (Table 1). Free monocarboxylic fatty acids, regardless of degree of saturation (0–5 double bonds) or chain length (C_14_ to C_22_) all decreased 35–75% in serum during the ischemic event, and then stayed low until the final timepoint at 24 h post injury. In contrast, the serum levels of four medium-chain (7–11 carbons) dicarboxylic acids increased significantly (2–3.6-fold) during the ischemic phase, and then returned to normal upon reperfusion (data not shown). Dicarboxylic fatty acids are produced from their monocarboxylic counterparts via a minor pathway of lipid metabolism known as fatty-acid omega oxidation, which initiates in the endoplasmic reticulum and then proceeds through the peroxisomal fatty-acid oxidation pathway [34]. Increased omega oxidation is normally associated with increased lipid load, such as during fasting or in diseases where mitochondrial fatty-acid oxidation is blocked [35]. The accumulation of dicarboxylic fatty acids noted during ischemia could, therefore, indicate either impaired mitochondrial or peroxisomal function during the phase of ischemia.

Some changes were also noted in lysophospholids (Supplementary Table S1). Lysophospholipids are phospholipids where one acyl chain has been removed by hydrolysis, and this could indicate membrane damage during ischemia. While the biological roles of lysophospholipids are complex and poorly understood, increased serum lysophospholipids have been linked to several disease states [36]. Here, we observed differential effects of ischemia–reperfusion depending upon the saturation of the lysophospholipid acyl chain. Specifically, we detected five species with unsaturated acyl chains (18–20 carbons in length, 2–4 double bonds) that increased about twofold during ischemia, and then fell to either normal or below normal levels during reperfusion. Lastly, one lysophospholipid species with a saturated acyl-chain (1-stearoyl-GPI) decreased during ischemia and stayed low throughout reperfusion, while two other saturated species containing palmitoyl acyl-chains (C_16:0_) were unaffected by ischemia but showed significantly elevated levels in serum after 24 h of reperfusion.

### 3.4. Polyunsaturated Fatty Acids (PUFAs) Are Suppressed by I–R Injury

**Figure 5:**
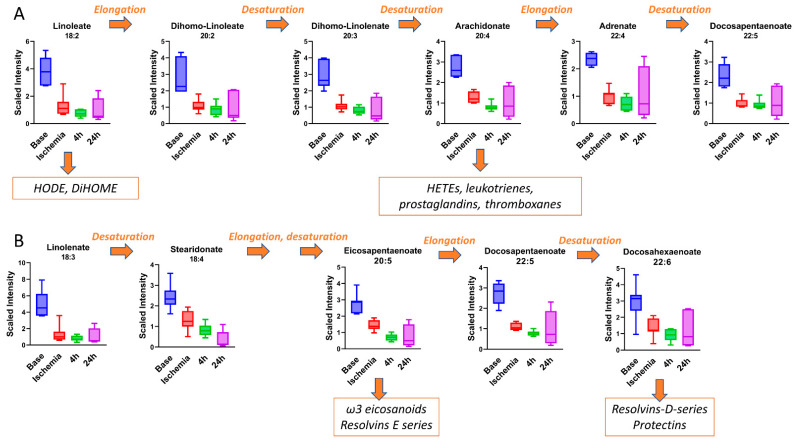
**I–R injury suppresses serum level of PUFAs.** (**A**) Pro-inflammatory N-3 PUFAs are synthesized through elongation and desaturation of linoleic acid (C_18:2_). (**B**) Anti-inflammatory N-6 PUFAs are synthesized through elongation and desaturation of alpha = linolenic acid (18:3). One-way ANOVA was run to determine significance, all are statistically significant. *N* = 7.

PUFA, particularly those of chain lengths C_20_ and C_22_, are key components of both cellular membranes and immune signaling pathways. There are two major series of PUFA, dubbed n-3 (omega-3) and n-6 (omega-6) depending upon the position of the double bonds. The n-3 series is derived through elongation and desaturation of the essential fatty acid linolenic acid (C_18:3_), while the n-6 series is derived from the essential fatty acid linoleic acid (C_18:2_). Many studies have established the cardioprotective effects of n-3 PUFAs, particularly EPA and DHA, while n-6 PUFAs are considered detrimental [37]. While the mechanisms behind this have not been fully established, the most invoked explanation is that downstream metabolites generated from n-3 PUFAs are anti-inflammatory (i.e., resolvins and protectins) while those generated from n-6 PUFAs are proinflammatory (HETE, eicosanoids, leukotrienes, prostaglandins, etc.). Interestingly, all C_20_/C_22_ PUFAs detected in our study, regardless of series, were significantly decreased in serum during the ischemic period, as were the essential fatty-acid precursors C_18:2_ and C_18:3_ (Figure 5A,B; Table 1). At present, the determinants of serum PUFA are not clear. One recent rodent study of synthesis and degradation kinetics suggested that it is the rate of catabolism that determines serum PUFA. The low serum PUFA following ischemia in pigs could be due to siphoning off C_20_ and C_22_ PUFA species for generation of second messenger lipid species such as eicosanoids and resolvins. Unfortunately, most of these signaling lipid species were not captured in our analysis. However, three species of the C_18_ signaling lipids HODE and DiHOME were detected, and all showed a similar degree of suppression to the PUFA species (Figure 5A). This suggests a potential role of these metabolites as biomarkers for the ischemic phase.

### 3.5. Ischemia Increases Serum Levels of N-Acetylated Amino Acids

Another interesting observation noted during ischemia was the increase in N-terminal acetylation (NTA) in many amino acids (AA). Metabolomic profiling demonstrated NTA of 19 AA and their metabolites (total of 27). Furthermore, eight of the top statistically significant 100 metabolites that showed an effect size of >0.5 were *N*-acetylated amino acids (Table 2). Of the 27, 18 of the metabolites showed significant changes during the ischemic period. While NTA increased in most amino acids, some decreased. Specifically, changes in *N*-acetyl leucine, isoleucine, methionine, and histidine accounted for the separation of the groups during ischemia. They were significantly increased, leading us to speculate that NTA could be a protective mechanism intended to lower BCAA, which can adversely affect cardiac function, or to shift away from FAO. NTA is considered generally irreversible and was discovered to be a degradation signal in a distinct branch of the N-end rule pathway, ubiquitin-mediated proteolysis. Although we cannot predict the impact of NTA on a target protein, a function for cytosolic NTA during stress responses has been proposed in plants. N-terminal imprinting of the proteome by the NatA complex has been proposed as an important switch for the control of metabolism, development, and cellular stress responses during drought stress [38]. While the role of acetylation of individual AAs in circulation still needs to be elucidated, the rise in these levels could be a potential biomarker for the ischemic phase. Like Ree et al., we propose that NTA could be a cellular surveillance mechanism during stress [39]. At the tissue level, the acetylation changes in the various proteins during ischemia are seen as protective through effects on metabolism [40], cell protection, and signal propagation. Whether such changes occur in cardiac tissue following IR needs to be established. *N*-Acetyl glutamate, *N*-acetyl serine, *N*-acetyl lysine, and *N*-acetyl glycine were decreased during ischemia; the decrease in *N*-acetyl glutamate and glycine may reflect a significant decrease in the total glutamate and glycine pool during IR.

### 3.6. Other Notable Changes in Amino-Acid Metabolism

**Figure 6:**
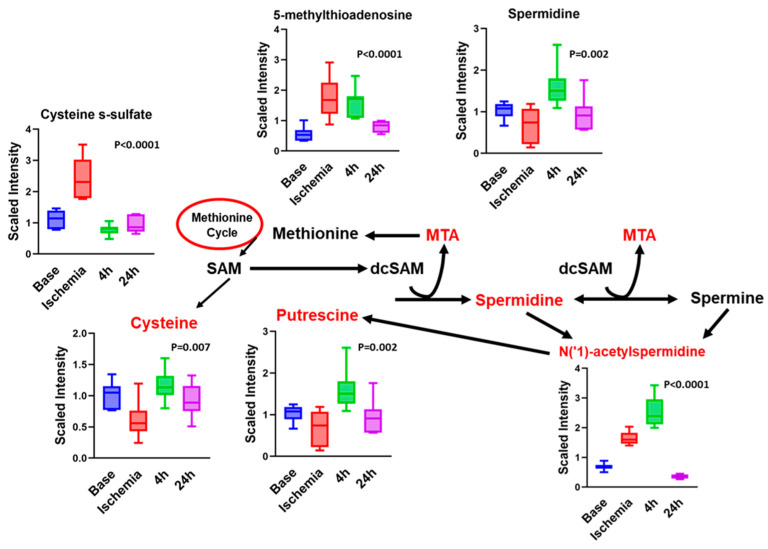
Significantly altered metabolites in the polyamine, methionine, cysteine, SAM, and taurine metabolism in serum induced by I–R. One-way ANOVA was run to determine significance. *N* = 7.

Notable changes included changes in metabolites in the polyamine, methionine, cysteine, *S*-adenosyl methionine (SAM), and taurine metabolism (Figure 6). The polyamine system is extremely sensitive to different pathological states and has been noted to be of importance in CNS injuries and many ischemia models. Spermidine and its derivatives influence cardiomyocyte apoptosis [41] and autophagy [42] during ischemia, and pharmacological polyamine depletion protects cardiomyocytes from ischemia-induced apoptosis [43]. We show that spermidine and *N*(‘1)-acetyl spermidine are significantly increased during ischemia (Table 3, Figure 6). Consistent with the increase in spermidine, putrescine levels tend to decrease with ischemia. *N*-Acetyl spermidine has been proposed as a biomarker for heart failure and has been associated with higher mortality in patients [44].

Tightly linked to the polyamine pathway are the methionine, cysteine, *S*-adenosyl methionine (SAM), and taurine metabolism pathways. There were no changes in serum methionine levels in our study. There was a notable reduction in cysteine levels during ischemia, while cysteine sulfate was elevated (Figure 6). Both returned to baseline with reperfusion. While SAM was not measured, SAH was decreased.

### 3.7. BCAA Metabolites and Glycerols Are Predominant during the Early Reperfusion Phase

**Figure 7:**
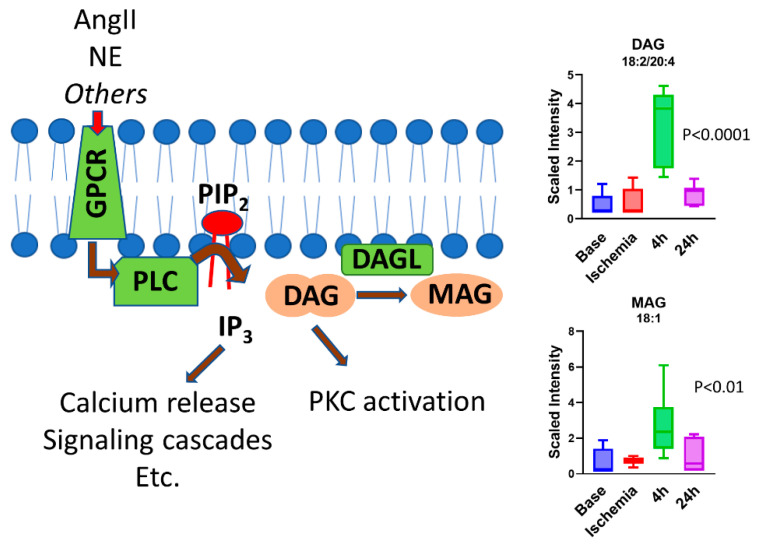
**DAGs and MAGs increase during early reperfusion (4 h) and drop back to baseline level at late reperfusion (24 h).** Shown are representative DAG and MAG species (see Supplementary Table S1 for full dataset). One-way ANOVA was run to determine significance. *N* = 7.

While the metabolomics profile at the end of ischemia reflects changes that occurred following ischemia, the profiles during reperfusion include changes that occurred following ischemia and reperfusion. From the metabolomics analysis performed at 4 and 24 h post reperfusion, we noted that some metabolite changes were specific to early (4 h) or late (24 h) reperfusion phases. One of the striking changes in metabolites noted in the early reperfusion phase was the accumulation of unusual intermediates of branched-chain amino-acid (BCAA) metabolism in the serum. These metabolites were the most prevalent and accounted for ~17% of the changes noted. Some of the unique BCAA metabolites are derived from the microbiome; the specific significance of these biochemical modifications in the context of ischemia–reperfusion needs to be studied. The other class of metabolites that was significantly increased during early reperfusion was represented by glycerols. Diacylglycerols (DAGs) are important lipid intermediates in triacylglycerol (TAG) biosynthesis and play a role as key signaling lipids. Here, we observed a strong, transient increase in several DAG species during early reperfusion (4 h), which became resolved by late reperfusion (24 h) (Figure 7). These DAG species were enriched in polyunsaturated acyl chains in the 1,2 positions, particularly C_18:2_ (linoleic) and C_20:4_ (arachidonic) acids. Because DAGs related to TAG synthesis typically contain mostly saturated acyl chains optimized for energy metabolism (i.e., C_16:0_, C_18:0_), the source of the elevated DAGs observed here was most likely the hydrolysis of plasma membrane phosphoinositides. A signaling pathway was previously well delineated [45] in which cardiac injury stimulates activity of phospholipase-C (PLC) at the plasma membrane, which hydrolyzes phosphoinositides such as phosphatidylinositol 4,5-bisphosphate (PIP2) into 1,2-DAG and inositol 1,4,5-trisphosphate (IP3). Both 1,2-DAGs and IP3 are powerful second messengers. IP3 induces the release of intracellular calcium stores with many downstream consequences. DAGs activate protein kinase C (PKC), which plays a significant role in mediating subsequent tissue injury in the heart [46]. Of note, only 1,2-DAGs, and not 2,3 or 1,3-DAG species, are capable of activating PKC [47]. Additionally, 1,2-DAGs can be further metabolized to either 1- or 2-monoacylglycerols (MAGs) by the enzyme DAG lipase. Indeed, in our study, the abundance of five unsaturated 1-MAG and 2-MAG species was observed to follow the same pattern as DAGs during reperfusion (Figure 7).

### 3.8. Bile Acids Are Key Metabolites during Late Reperfusion Phase

**Figure 8:**
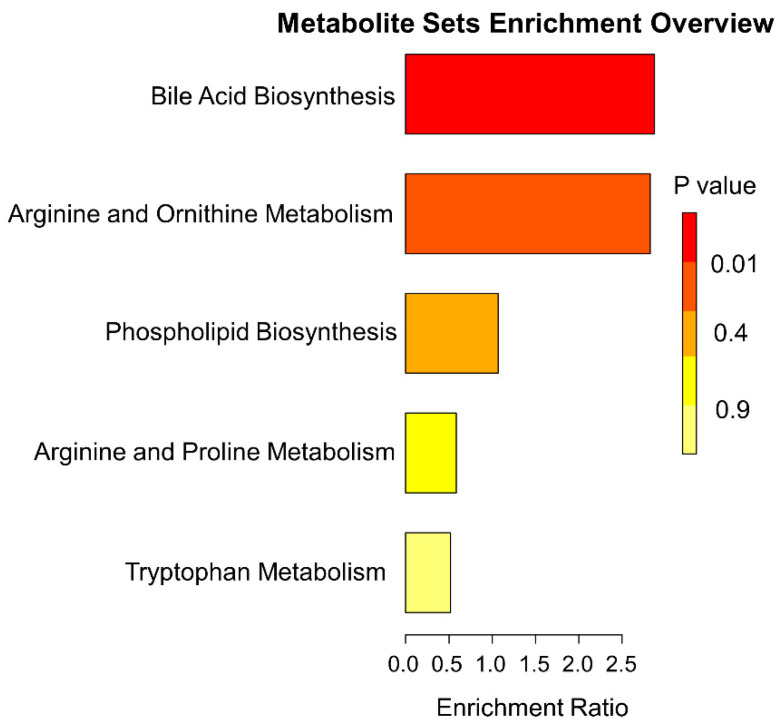
**Enrichment of bile acids and amino-acid metabolites during late reperfusion (24 h).** The functional analysis for metabolites from the late reperfusion module was performed using the Small Molecule Pathway Database (SMPDB) annotation system from MetaboAnalyst.

**Figure 9:**
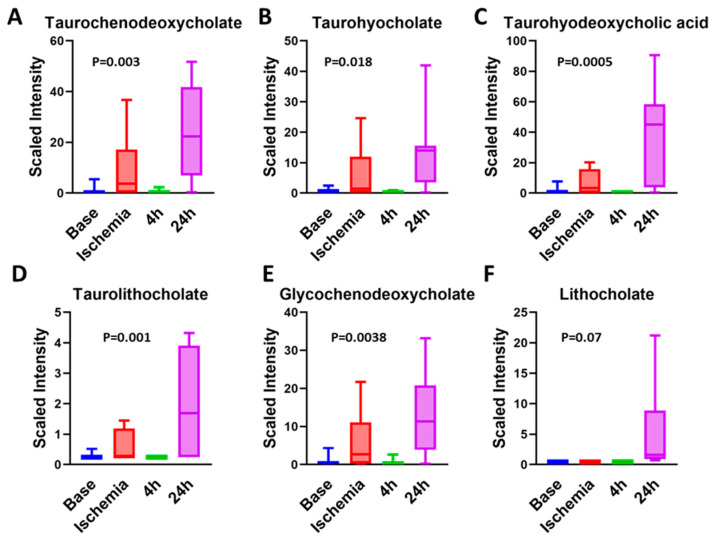
**Metabolites in primary and secondary bile acid synthesis increase during ischemia and late reperfusion.** Taurochenodeoxycholate (**A**), taurohyocholate (**B**), taurohyodeoxycholic acid (**C**), taurolithocholate (**D**), and glyochenodeoxycholate (**E**) were induced by both ischemia and late reperfusion, whereas lithocholate (**F**) was only induced by late reperfusion. One-way ANOVA was run to determine significance. *N* = 7.

One of the key groups of metabolites that marked the late reperfusion phase was represented by the primary and secondary bile acids (Figure 8 and Figure 9). Primary and secondary bile acids increased during the ischemic phase by 2–10-fold, and they decreased significantly during early reperfusion, but increased significantly during late reperfusion phase, with levels reaching as high as 80-fold higher than those observed during early reperfusion (Table 4). Liver enzymes (ALT, AST) were also significantly increased at 24 h (AST of 5920 IU/L and ALT of 544 IU/L compared to AST of 15–53 IU/L and ALT of 20–48 IU/L reported in healthy miniature swine (Yucatan, Hanford minipig)), indicating significant liver ischemia [48]. The elevated secondary bile acids could reflect bowel microbiota and perfusion during the reperfusion phases.

Bile acids have recently attracted attention for their effects on the heart through farnesoid X receptor (FXR), TRG5 receptors, and muscarinic receptors. Various bile acids use different receptors. For example, chenodeoxy cholic acid (CDCA), deoxycholic acid (DCA), and lithocholicacid (LCA) use FXR and TRG5 for signaling, while ursodexoycholic acid (UDCA) uses only TRG5 for signaling. Activation of FXR and TRG5 has been shown to slow the atheroma formation and decrease plaque size and inflammation [49]. Lower fasting serum total bile acids levels (including total levels of cholic acid, CDCA, DCA, and UDCA) are independently associated with severe coronary artery disease and MI [32], and they have been proposed as a biomarker to predict the presence and severity of coronary artery disease. Feng et al. showed that, in a select group of patients. i.e., menopausal women with type 2 diabetes mellitus, myocardial infarction and coronary artery disease were negatively correlated with total serum bile acids [50]. Consistent with the above, Huang et al. showed that increased admission serum total bile acids could be associated with decreased 3 month mortality in patients with acute ischemic stroke [51]. However, in asymptomatic individuals, Zhang et al. showed that higher serum total bile acid level was associated with the severe coronary artery stenosis and high-risk coronary artery plaques [52].

The effects of bile acids on the cardiac function are based on the hydrophilicity of the bile acid, with hydrophobic bile acids being more cardiotoxic. LCA is the most hydrophobic bile acid; hence, only a small amount of LCA is usually reabsorbed back into the enterohepatic circulation. However, in our porcine I–R model, the levels of LCA increased 12-fold during late reperfusion compared to early reperfusion (Table 4). The least toxic bile acid is UDCA. UDCA, synthesized by dehydroxylation of the free bile acid CDCA, is the most hydrophilic bile acid and has been shown in many studies to be cardioprotective. In rodent models of I–R, UDCA was shown to improve contractile function during reperfusion, inhibit the opening of the mitochondria permeability transition pore (MPTP) and Bcl-2 via the PI3K/Akt pathway, and improve endothelium- and NO-independent vasodilatation, maintaining the arterial flow in patients with heart failure. Unfortunately, our metabolic analytes profile did not include UDCA. The alterations in bile acids are similar to what has been observed in humans following cardiac arrest and resuscitation [53]. The taurine conjugates of bile acids were found to be increased in humans, compared to a decrease in mice.

### 3.9. Serum Plasmalogens Increase during the Reperfusion Phase

**Figure 10:**
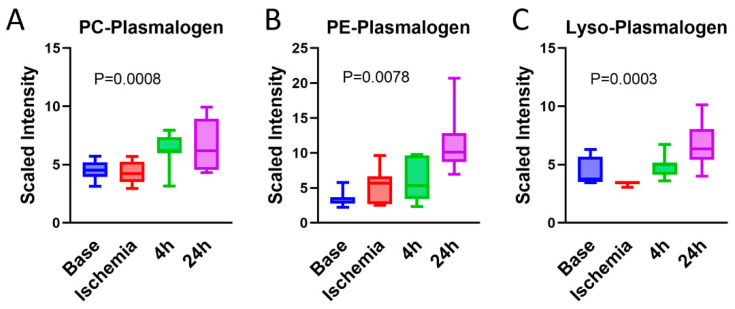
**Serum plasmalogens increase during the reperfusion phase**. Plasmalogen species were grouped by class and combined. Phosphatidylcholine (PC) plasmalogens (**A**), phosphatidylethanolamine (PE) plasmalogens (**B**), and lysoplasmalogen (**C**) were all increased with reperfusion. One-way ANOVA was run to determine significance. *N* = 7.

Plasmalogens are primarily synthesized by the liver and circulated to the periphery in the blood as a component of lipoproteins. Synthesis of plasmalogens involves initial steps in the peroxisome followed by completion in the endoplasmic reticulum. Phosphatidylcholine (PC) plasmalogen species, with a choline head group, are a minor fraction of total plasmalogens but are enriched in the heart [54], while phosphatidylethanolamine (PE) plasmalogens are found throughout the body. Exogenous plasmalogens administered prior to ischemic injury are cardioprotective [55]. Here, we observed increasing serum PC and PE plasmalogens with increasing reperfusion time (Figure 10), likely reflecting increased synthesis by the liver to replace membrane plasmalogens depleted by the cardiac injury. During cardiac ischemia, it is believed that phospholipases specific for membrane plasmalogens are activated, which cleave acyl chains from plasmalogens and release lysoplasmalogens containing only one acyl-chain [56]. Indeed, lysoplasmalogens increased with reperfusion in our model (Figure 10). Depletion of plasmalogens from the plasma membrane has been shown to stimulate biosynthesis of new plasmalogens [57]; thus, increased degradation during the ischemic period due to phospholipases may trigger biosynthesis during reperfusion.

## 4. Strengths and Limitations

This study had several unique strengths and some limitations. Studies were performed in pigs that were young, healthy, and without comorbidities; therefore, the metabolic profile was a clear reflection of the phases of I–R. The metabolites offered unique insights into the pathophysiological changes in the various phases and helped segregate the various phases: ischemia, early reperfusion, and late reperfusion. Changes in the metabolites during ischemia reflected the ischemic phase only, while analysis in other phases included the changes seen in ischemia along with early and late reperfusion. Nevertheless, we found interesting metabolic shifts in each of the reperfusion phases. The metabolomic analysis conducted in serum offered the advantage of being more practical and translatable. While these were some of the strengths, there were also some limitations. The serum metabolites reflected not only the changes from cardiac tissue but also those changes related to poor perfusion. Future studies should compare the changes in serum metabolomics to changes in cardiac tissue metabolomics. No gender differences were analyzed in the present study. Furthermore, since the pigs were otherwise healthy, the effects of various comorbidities that exist in patients who experience MI and, therefore, could affect serum metabolites were not studied. A much larger sample size will be needed to assess changes with various phases in humans as those who experience MI have multiple comorbidities that could affect the metabolic profile. Studies in humans have focused on serum levels of key metabolites that could provide prognostic information. While these are important, changes in metabolite profiles during I–R are dependent on the baseline status of these key metabolites.

**In conclusion**, metabolomics in young, healthy pigs with no comorbidities offered a unique insight into the changes in serum metabolites during the different phases. These findings need to be leveraged in the future toward the identification of biomarkers and prognostic markers that can be translated to clinical practice.

## Figures and Tables

**Table 1:** **List of fold changes of FFAs during ischemia and reperfusion phases**.

	Ischemia	4 h	24 h	*p*-Value	Effect Size
**Saturated Fatty Acids**					
Myristate (14:0)	0.29	0.22	0.24	1.4 × 10^−11^	0.67
Pentadecanoate (15:0)	0.64	0.53	0.57	5.5 × 10^−11^	0.65
Palmitate (16:0)	0.44	0.30	0.31	6.5 × 10^−11^	0.66
Margarate (17:0)	0.46	0.29	0.27	1.0 × 10^−11^	0.67
**Monounsaturated Fatty Acids**					
Palmitoleate (16:1n7)	0.25	0.15	0.18	2.0 × 10^−12^	0.69
10-Heptadecenoate (17:1n7)	0.25	0.14	0.16	2.0 × 10^−12^	0.70
Oleate/vaccenate (18:1)	0.40	0.22	0.22	3.4 × 10^−11^	0.72
10-Nonadecenoate (19:1n9)	0.36	0.24	0.28	1.0 × 10^−10^	0.65
**Polyunsatured Fatty Acids**					
Linoleate (18:2n6)	0.34	0.19	0.24	7.0 × 10^−12^	0.69
Linolenate (alpha or gamma; (18:3n3 or 6))	0.27	0.15	0.20	8.0 × 10^−12^	0.65
Mead acid (20:3n9)	0.36	0.21	0.21	3.7 × 10^−11^	0.58
Dihomo-linolenate (20:3n3 or n6)	0.37	0.27	0.26	1.2 × 10^−10^	0.68
Arachidonate (20:4n6)	0.47	0.30	0.36	6.6 × 10^−10^	0.70
Eicosapentaenoate (EPA; 20:5n3)	0.51	0.25	0.25	1.0 × 10^−11^	0.66
Adrenate (22:4n6)	0.44	0.32	0.44	1.0 × 10^−7^	0.61
Docosapentaenoate (n3 DPA; 22:5n3)	0.41	0.29	0.37	1.5 × 10^−9^	0.66

Fold change denote a decrease from baseline.

**Table 2:** **Change of serum levels of *N*-acetylated amino acids during ischemia phase**.

Metabolite Name	F-Statistic	Effect Size	Changes with Ischemia	*p*-Value
*N*-Acetyl serine	10.755	0.59	Decreased	0.0088
*N*-Acetyl histidine	11.438	0.73	Increased	0.0215
*N*-Acetyl valine	22.487	0.78	Increased	0.0015
*N*-Acetyl leucine	14.858	0.69	Increased	0.0002
*N*-Acetyl isoleucine	8.821	0.57	Increased	0.0143
*N*-Acetyl methionine	31.556	0.97	Increased	0.0462
*N*-Acetyl phenylalanine	19.688	0.71	Increased	0.0000
*N*-Acetyl tryptophan	31.036	0.77	Increased	0.0001

**Table 3:** **Change in serum levels of metabolites in spermidine metabolism during ischemia phase**.

Metabolite_Name	F-Statistic	Effect Size	Changes with Ischemia	*p*-Value
*N*(‘1)-Acetyl spermidine	88.846	0.912	Increased	2.37812 × 10^−5^
Spermidine	15.366	0.631	Increased	8.84902 × 10^−5^
5-Methylthioadenosine (MTA)	15.348	0.609	Increased	0.000146238

**Table 4:** **List of fold change of metabolites in bile acid metabolism during ischemia and reperfusion phases**.

	Biochemical Name	45 min	4 h	24 h
Primary bile acid metabolism	Glycochenodeoxycholate	**6.46**	**0.61**	**13.60**
	Taurochenodeoxycholate	**8.39**	**0.52**	**21.49**
Secondary bile acid metabolism	Deoxycholate	**0.77**	**0.74**	**1.55**
	6-beta-Hydroxylithocholate	**0.48**	**0.29**	**11.03**
	Lithocholate	**1.00**	**1.00**	**12.05**
	Glycolithocholate	**2.63**	**0.74**	**11.33**
	Glycolithocholate sulfate *	**1.19**	**0.91**	**4.68**
	Taurolithocholate	**1.99**	**0.86**	**7.45**
	6-Oxolithocholate	**0.36**	**0.22**	**7.41**
	Hyocholate	**1.06**	**0.46**	**7.09**
	Glycohyocholate	**4.17**	**0.24**	**7.80**
	Taurohyocholate *	**10.22**	**0.49**	**20.89**
	Hyodeoxycholate	**0.76**	**0.57**	**20.39**
	Taurohyodeoxycholic acid	**4.47**	**0.33**	**26.68**
	glycohyodeoxycholate	**3.08**	**0.34**	**12.54**

Dark green denotes a decrease from baseline, while red denotes an increase from baseline (*p* < 0.05), *-indicates a compound that has not been confirmed based on a standard, but Metabolon is confident in its identity.

## Data Availability

Original data appears in Table S1 of the Supplement.

## Supplementary Materials

The following supporting information can be downloaded at: https://www.mdpi.com/article/10.3390/ijms23126711/s1.
