# Supplementary material for: Serum Metabolomics Reveals Distinct Profiles during Ischemia and Reperfusion in a Porcine Model of Myocardial Ischemia–Reperfusion
Source: Int J Mol Sci. 2022 Jun 16;23(12):6711. doi: 10.3390/ijms23126711 (PMC9223436; doi:10.3390/ijms23126711)
Supplement: Supplementary file 1 [file ijms-23-06711-s001.zip › Supplemental Figure S1.pdf]

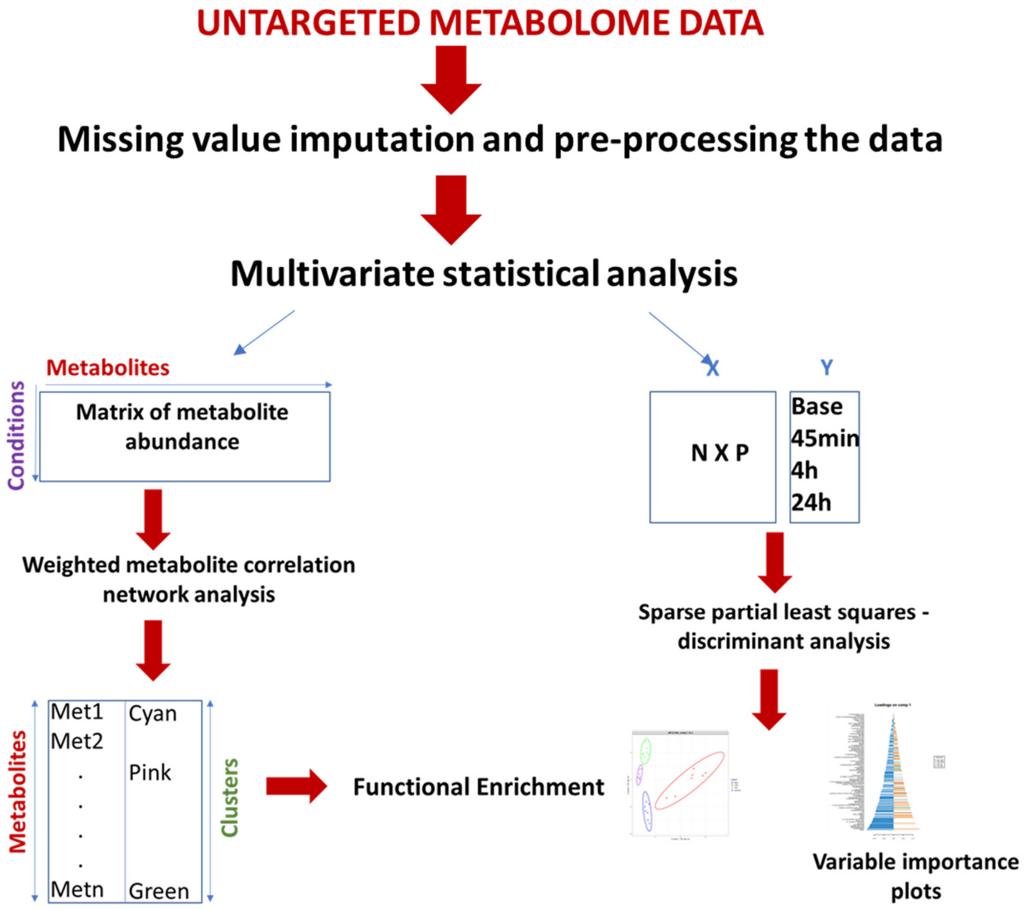

Supplemental Figure S1. Overview of the metabolome data analysis. The data was pre-processed (scaled), and rows with all zero values removed for further analysis. Downstream analysis includes (1) co-expression analysis of the metabolites wherein we constructed the weighted metabolite correlation networks with R packages, and the metabolites were clustered into modules. The module eigenmetabolites of each module were then correlated to the phenotype, and the modules were further characterized using SMPDB pathway annotation., and (2) sparse-partial least squares discriminant analysis (sPLS-DA) where in the metabolite matrix is represented as X ('N'-samples measured across 'P' conditions), and Y is a factor of the conditions. sPLS_DA was implemented for classification and variable-selection in a one-step procedure and visualized using sample and variable importance plots.
